# Review on Desirable Microbial Phytases as a Poultry Feed Additive: Their Sources, Production, Enzymatic Evaluation, Market Size, and Regulation

**DOI:** 10.1155/2024/9400374

**Published:** 2024-06-07

**Authors:** Olyad Erba Urgessa, Rufael Koyamo, Hunduma Dinka, Ketema Tafess, Mesfin Tafesse Gemeda

**Affiliations:** ^1^School of Biological Sciences and Biotechnology, College of Natural and Computational Sciences, Haramaya University, Dire Dawa, Ethiopia; ^2^Department of Applied Biology, School of Applied Natural Science, Adama Science and Technology University, Adama, Ethiopia; ^3^Department of Biology, College of Natural and Computational Sciences, Oda Bultum University, Chiro, Ethiopia; ^4^Institute of Pharmaceutical Science, Adama Science and Technology University, Adama, Ethiopia; ^5^Biotechnology and Bioprocess Center of Excellence, Addis Ababa Science and Technology University, Addis Ababa, Ethiopia

## Abstract

Poultry's digestive tract lacks hydrolytic phytase enzymes, which results in chelation of dietary minerals, vital amino acids, proteins, and carbohydrates, phytate-phosphate unavailability, and contamination of the environment due to phosphorus. Therefore, it is necessary to use exogenous microbial phytases as feed additive to chicken feed to catalyze the hydrolysis of dietary phytate. Potential sources of microbial isolates that produce desired phytases for chicken feed supplementation have been isolated from agricultural croplands. It is achievable to isolate phytase-producing bacteria isolates using both broth and agar phytase screening media. Potential substrates for submerged fermentation (SmF) for bacterial phytase production and solid-state fermentation (SSF) for fungal phytase production include rice and wheat bran. Following fermentation, saturated ammonium sulphate precipitation is typically used to partially purify microbial culture filtrate. The precipitate is then desalted. Measurements of the pH optimum and stability, temperature optimum and stability, metal ions stability, specificity and affinity to target substrate, proteolysis resistance, storage stability, and in vitro feed dephosphorylation are used to perform an enzymatic evaluation of phytase as an additive for poultry feed. The growth of the feed phytase market is primarily due to the expansion of chicken farms to meet the demand for meat and eggs from humans. The Food and Drug Administration in the USA and the European Food and Safety Authority are primarily in charge of putting rules pertaining to feed phytase use in chicken feed into effect. Conclusively, important components of the production of phytase additives for poultry feed include identifying a reliable source for potential microbe isolation, selecting an economical method of phytase production, thoroughly characterizing the biochemical properties of phytase, and comprehending the size and regulation of the current feed phytase market.

## 1. Background

Phytases have attracted considerable attention in the area of animal nutrition, environmental protection, and biotechnology [1, 2]. In poultry feed, nearly 80% of the total phosphorus content is stored as phytate. Unfortunately, the gastrointestinal tracts (GIT) of poultry exhibit little to no phytase activity [3]. Therefore, from the perspective of phosphate bioavailability, phytic acid and its salts, phytates, are considered nutritionally inactive substances. Phytic acid or phytate chelate vital nutrients, carbohydrates, proteins, and essential amino acids [4, 5]. Furthermore, in regions with intensive livestock production, consuming large amounts of feed high in phytic acid or phytate by monogastric animals leads to phosphorus-related environmental pollution, such as algal blooms [6]. Thus, exogenous microbial phytase has been used as monogastric animals' feed additive because the phytase catalyzes hydrolysis of phytic acid improving nutrient bioavailability and uptake and reducing pollution caused by fecal excretion of phosphorus [7, 8].

Microbes that produce phytase have been selected from a variety of sources. Phytase-producing filamentous fungi have been reported to be isolated from soil [9], citrus pulp pellets, wheat and rice bran [10], and poultry farms [11]. There have been reports of using poultry farms, rhizospheric soils, compost and degraded wood [12], indigenous fermented cheese product [13, 14], and the soil surrounding the maize field (halosphere), the soil surrounding the maize root (rhizosphere), and inside the maize root (endophyte) as sources to isolate phytase-producing bacteria [15, 16]. Researchers have avoided false positive halo zones on PSM agar plates when screening for phytase-producing bacteria by first flooding the plate with cobalt chloride solution, then replacing the solution with freshly made coloring reagent (a mixture of aqueous ammonium molybdate and ammonium vanadate), and lastly removing the coloring reagent [13, 15, 17]. Subsequently, the isolate with the highest phytase activity in the broth or the best halozone formation on the agar plate is chosen. 16S rRNA gene sequencing is often used to identify the chosen phytase-producing bacterial isolate molecularly [18], whereas Internal Transcribed Spacer (ITS1, 5.8 S, ITS2) region sequencing is typically used for the molecular identification of the chosen phytase-producing fungal isolate. Molecular tools that supplement morphological characterization for fast and accurate fungal identification are highly promising [19].

A selected microbial strain that produces phytase has been used to produce phytase through submerged fermentation (SmF) or solid-state fermentation (SSF) of agricultural and agroindustrial waste materials. SSF is the common fermentation type in phytase production from fungal strain [17]. On the other hand, SmF has frequently been employed for producing bacterial phytases [20]. Microbial culture filtrate is harvested as crude phytase after fermentation, and the obtained crude phytase is partially purified by precipitating with saturated ammonium sulphate and then desalting the resultant precipitate. Several researchers documented the production of crude phytase [11, 21–26] and partially pure phytase [12, 13, 27–29] with two or more desirable properties for supplementing poultry feed.

In order to establish the novel phytase as a feed additive for nutritional enhancements, extensive biochemical characterization and dephytinization activity of the phytase have been conducted [11]. For the purpose of enzymatically evaluating phytase as a poultry feed additive, specific factors such as pH optimum and stability, temperature optimum and stability, metal ions stability, specificity and affinity to target substrate, proteolysis resistance, storage stability, and in vitro feed phytate dephosphorylation have been taken into consideration. Harvesting crude phytase from native microbial culture filtrate or partially purifying the crude phytase is a feasible method of using it as an additive in poultry feed in developing countries [30]. Accordingly, the sources, isolation, and production of phytase with one or more biochemical characteristics resembling the chicken gut conditions or feed processing conditions are covered in this review. In addition, the review discusses market size and regulations concerning phytase additives in poultry feed.

## 2. Sources of Desirable Phytase as Poultry Feed Additive

Monogastric animals are known to have little to no phytase activity in the GIT [6, 9], necessitating the addition of exogenous microbial phytases to feed in order to catalyze the hydrolysis of dietary phytate [31]. The major sources of exogeneous phytase are fungal and bacterial strains [20, 32]. While native plants and animals are also sources of exogenous phytase [33], native algae as a source is hardly available. This is a significant gap since almost all phytase studies noted in the background that phytate, which is expelled by monogastric animals, is what causes algal blooming [6]. If algae are unable to use phytate effectively, which necessitates the synthesis of phytase, the bloom cannot occur. Nonetheless, it has been documented that transgenic microalgae have been developed as a source of phytase for use as a feed additive in monogastric animals [4, 34].

Phytase-producing native fungi and bacteria have been screened from various source materials. The sources of the best phytase-producing isolates are presented in Table 1. *Woodfordia fruticosa* dried flower buds were utilized as a source material to isolate intracellular phytase-producing yeast, *Pichia anomalai* UDDM-55 [37]. Composting soil [9], corn, citrus pulp pellets, wheat, and rice bran [10], soil from the top layer of agriculture croplands, including maize (*Zea mays*), wheat (*Triticum aestivum*), black gram (*Vigna mungo*), and rice (*Oryza sativa*), and poultry farms [11] were used as source materials to isolate filamentous fungi. Poultry farms, rhizospheric soils, compost and degraded wood [12] and halosphere (soil surrounding the maize field), rhizosphere (soil surrounding the maize root), and endophyte (inside the root) [15, 16] were used as sources to isolate bacteria. Conventionally fermented cheeses, such as Turkey's Lor cheese [14] and India's Kalari [13], have also been identified as sources of bacteria that produce phytase. It is recommended to isolate probiotic bacteria that produce phytase from dairy products because these bacteria have been classified as safe (GRAS) by the Food and Drug Administration (FDA) [13]. Kali and Kudithi, two more traditionally fermented foods, have been identified as sources of both yeasts and bacteria that produce phytase [15]. Indian rural women use Kali, which is the leftover water from traditionally fermented rice, to cook cereal meals. Kudithi, on the other hand, is fermented liquid cattle feed.

## 3. Isolation and Screening of Phytase-Producing Microbe

**Figure 1:**
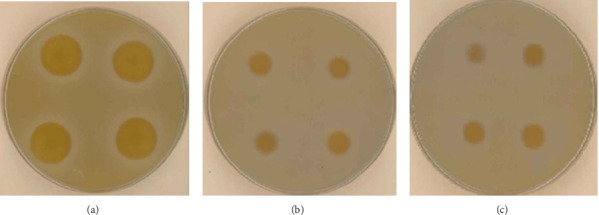
Clear zones formed by phytase activity on the phytase screening agar plates. Clear zone of phytase of (a) *Aspergillus ficuum* (NRRL 3135), (b) *Lactobacillus plantarum* (B-4496), and (c) *Lactobacillus acidophilus* (B-4495). Each inoculum (20 *μ*l = 10 × 10^6^ CFU/mL) was inoculated on agar plates using point inoculation, and plates were incubated at 37°C for bacteria and 30°C for fungus for 2 days. Then, all plates with clear zones were washed with distilled water to remove microorganisms. Then, plates were covered with 2% cobalt chloride solution and incubated for 5 min at room temperature. Then, the cobalt chloride solution was discarded, and the plates were covered with an equal volume of freshly prepared 6.25% ammonium molybdate and 0.42% ammonium vanadate mixture. After 5 min incubation at room temperature, the solution was discarded and clear zones were still present [17].

Both qualitative and quantitative techniques were used in the isolation and screening of the phytase-producing microorganisms. The qualitative approach is based on the largest clear zone formation on the PSM agar plate (Figure 1). PSM includes sodium phytate as a solitary source of phosphorus [13], calcium phytate as the only source of phosphorus [12, 23, 28], or sodium phytate as sources of both carbon and phosphorus [1, 10, 21, 39]. If phytate is the only source of carbon and phosphorus, fungal species that can grow on these PSM agar plates must create an extracellular phytase to use the carbon and phosphorus found in phytate [10]. The constituents of commonly used PSM are presented in Table 2.

Unfortunately, false positive reactions (zones of clearing around microbial colonies) can be seen for acid-producing bacteria, which means that PSM lacks specificity. Because of the dissolution of phytate/phytic acid at low pH levels brought on by acid production, a clear zone forms. To address this issue, a two-step counterstaining procedure was created [40]. Agar plates are first flooded with cobalt chloride solution, which is done on the grounds that it causes the halo zone to vanish by reprecipitating acid-solubilized phytate. The second step is to decant the cobalt chloride solution. This is followed by adding the coloring reagents, aqueous molybdate, and ammonium vanadate solution, based on the idea that a positive reaction will develop a yellow color as a result of the reaction between the reagent and the released phosphate from phytate hydrolysis. Numerous researchers have employed this technique [13, 15, 17].

Using a quantitative method, isolates are cultivated in PSM broth and their highest level of phytase activity is assessed (Table 1). Certain strains that are identified as having the ability to produce phytase on an agar plate are unable to do so in broth. Furthermore, some strains that test negative on an agar plate yield high levels of phytase in broth [15]. Consequently, the best phytase-producing microbes have been identified using both the broth and the agar plate [11–13, 15] (Table 1).

## 4. Identification of Phytase-Producing Microbe

Species-level identification of the chosen isolates comes next, following the screening and selection of the best phytase-producing microbes. 16S rRNA gene sequencing is typically used for the molecular identification of bacteria in general and phytase-producing bacteria in particular [18, 38]. In one study, 16S rRNA gene sequencing was used to identify the chosen phytase-producing bacterial isolates after microscopic inspection [12]. Bhagat et al. [13] used only 16S rRNA gene sequencing in the study to identify the chosen phytase-producing bacterial isolate (Table 1). According to Johnson et al. [18], sequencing the full-length (∼1500 bp) 16S rRNA gene allows for taxonomic resolution at the species and strain level, which cannot be obtained by targeting 16S variable regions (V1, V2, V3, V4, V5, V6, V7, V8, and V9) with short-read sequencing platforms. Moreover, Durazzi et al. [41] demonstrated that, provided a sufficient number of reads is available, whole genome shotgun sequencing is more powerful than 16S rRNA gene sequencing at identifying less abundant taxa.

Internal Transcribed Spacer Region (ITS1, 5.8 S, ITS2) sequencing is typically used for the molecular identification of phytase-producing fungal isolates [19]. Alves et al. [23] identified the selected fungi using ITS region sequences. Besides, 18S rRNA gene sequencing is also used for the identification of the highest phytase-producing fungal isolate [11] (Table 1). The entire ITS region, as well as the ITS1 and ITS2 subregions, is frequently utilized for fungal identification; however, the most successful identification method was one that relied on the entire ITS region [42]. Molecular tools that supplement morphological ones show great promise for fast and accurate species-level fungal identification [19].

## 5. Phytase Production

Phytase has been produced by submerged, solid, or semisolid-state fermentation. The production of phytase from fungal strains frequently involves solid-state fermentation (SSF) [17]. A better phytase production was reported from SSF than from SmF by fungal strains [43]. SSF, as opposed to submerged fermentation (SmF), improves enzyme secretion with ease of recovery and purification, uses less expensive substrates, such as agri-waste, and requires less water. It also stimulates fungal growth with fewer nutrient requirements [32]. However, SmF can be scaled up very easily for commercial phytase productions, and stirring during the SmF process ensures homogeneity in the phytase properties [17]. SmF has been widely used in bacterial strain phytase production [20]. In fact, *B*. *subtilis* US417 has been reported to produce more phytase on wheat bran SmF (112 U/g of WB) than on SSF (85 U/g of WB) [44]. Furthermore, when temperatures are high enough to dry out media and lower water activity below the required level for microbes, SSF is ineffective for producing enzymes. Semisolid-state fermentation (semi-SSF) is a preferable choice in this situation [45].

Because it may lower the cost of producing enzymes and result in a less expensive final product, the use of agricultural and agroindustrial waste substrates in phytase production has been widely reported [46]. The potential substrates, microbial strains, and types of fermentation along with the amount of produced phytase are shown in Table 3. From affordable substrates SSF such as coconut oil cake by *Rhizopus oligosporus* [61] and citric pulp bran by *A. niger* FS3 [51], phytase production of 14.29 U/gds and 0.62 U/mL, respectively, was reported. The production of 3.91 U/mL of phytase by *A. niger* F3 from citrus peel SmF was also reported [50].

One frequently utilized agroindustrial residue for the production of phytase is wheat bran (Table 3). It has nutrients that microbes need, including minerals (Ca, K, Mg, and Fe), B vitamins (thiamin, niacin, riboflavin, and folate), insoluble fiber, essential fatty acid, starch, protein, and phytic acid [67]. Wheat bran can, therefore, induce and promote the production of phytase [52, 58]. Maximum phytase production (94 U/mL) was obtained with wheat bran SmF by *Klebsiella* sp compared to the SmF of rice bran and chickpea by the same bacterial strain [58]. From wheat bran SSF by *Schizophyllum commune* [26] and by *A. niger* NT7 [11], the highest phytase production of 113.7 U/gds and 208.30 ± 0.22 U/gds, respectively, was reported. A study showed the production of a higher mount of phytase from concentrated (upto 60%) wheat bran extract SmF. Furthermore, the utilization of wheat bran powder resulted in a higher level of phytase than that of wheat bran extract utilization [65].

Because rice bran contains proteins, fats, carbohydrates, and minerals, it has found extensive use in the bioprocess industry to produce phytase (Table 3). *R. oligosporus* MTCC556 produced 31.3 U/gds of phytase from rice bran SSF, according to Suresh et al. 2016 [62]. In comparison to wheat bran and oat bran SmF by *Aspergillus flavus* PHY168, Ahmed et al. 2018 [47] reported the highest phytase production from 5% rice bran SmF by the strain. Furthermore, it was reported that *Thermoascus aurantiacus* SL16W produced more phytase from rice bran Sem-SSF than from wheat bran Sem-SSF [45]. Based on the aforementioned research, it is unclear whether rice bran or wheat bran promotes the production of every microbial phytase more effectively.

## 6. Partial Purification of Phytase

**Figure 2:**
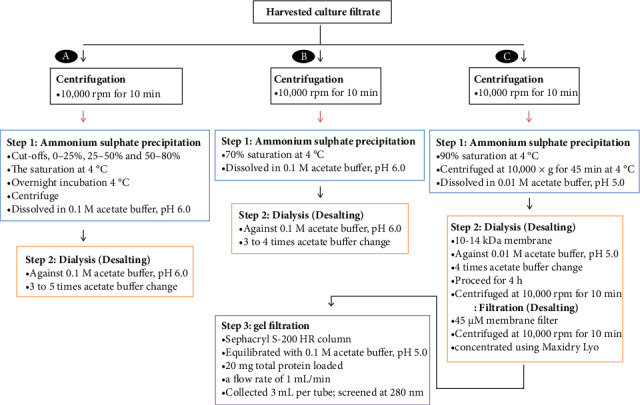
Phytase partial purification steps: (a) phytase of *Humicola nigrescens* BJ8 was extracted by mixing 50 mL of distilled water containing 0.1%Tween-80 with solid-state fermented substrate. The mixture was shaken at 200 rpm for 60 min and filtered through a double-layer muslin cloth. The filtrate obtained was centrifuged and administered to ammonium sulphate precipitation followed by desalting [28]. (b) Culture broth of *Serratia* sp. PBS-15 and *E. cloacae* PBS-45 was centrifuged and administered to ammonium sulphate precipitation followed by desalting [12]. (c) The spent culture media obtained from *A. foetidus* MTCC 11682 culture were harvested, filtered, and the filtrates were subjected sequentially to ammonium sulphate precipitation, desalting, and gelfiltration [29].

The production of crude phytase with two or more desirable properties for supplementing poultry feeds has been reported by several researchers [11, 21–26]. In addition, a number of researchers have reported the production of partially pure phytase [12, 13, 27–29]. In the context of production and characterization of phytase, purification has been considered as partial purification and purification. It has been widely documented that in partial purification, saturated ammonium sulphate salting out is followed by desalting procedures (Figure 2). Ammonium sulphate precipitation is the most popular and straightforward technique for partially purifying phytase from microbial sources, according to Bala et al. [28]. It is interesting to note that adding fungal desalted phytase precipitated by ammonium sulphate to broiler feed greatly increased P utilization and decreased P excretion into the environment [29].

When purifying proteins, such as enzymes, the effectiveness of the purification processes is typically assessed based on specific activity, purification fold, and percent yield (recovery), which can be calculated using the following equations:

(1) $$\begin{array}{c} \text{Specific activity}=\left(\frac{\text{the activity of phytase at each steps}}{\text{Total protein content of respective steps}}\right), \end{array}$$

(2) $$\begin{array}{c} \text{Purification fold}=\left(\frac{\text{specific activity of phytase at each steps}}{\text{specific activity of phytase at initial step}}\right), \end{array}$$

(3) $$\begin{array}{c} \%\text{Yield}=\left(\frac{\text{phytase activity of each steps}}{\text{phytase activity of initial step}}\right)\times 100. \end{array}$$

Partial purification of steps of microbial phytase with suitable properties for poultry feed supplementation and the efficacy of parameters are shown in Table 4. Phytase of *A. niger* NCIM 563 was partially purified by ammonium sulphate precipitation (95%) followed by Sephadex G-25 column-based desalting. Specific activity of 224 FTU/mg, 3.0 purification fold, and 95% purification yield were obtained from the partial purification [68]. Partial purification of *H. nigrescens* BJ8 phytase with 20.55 fold and 96.71% yield [28], and crude phytase of *A. foetidus* MTCC 11682 with 23.4 fold and 12.9% yield [29] were reported. Purification fold and yield for partial purification of bacterial phytase were reported to be 2.18 and 44.63% for *Serratia* sp. PSB-15 phytase, and 2.55 and 62.30 for *E. cloacae* PSB-45 phytase [12]. In a different investigation, Bhagat et al. [13] reported that the extracellular phytase of *P. acidilactici* SMVDUDB2 had been partially purified with a 6.42 fold yield.

## 7. Phytase Enzymatic Evaluation as a Poultry Feed Additives

For the enzymatic evaluation of phytase as a poultry feed additive, factors such as pH optimum and stability, temperature optimum and stability, metal ions stability, specificity and affinity to target substrate, proteolysis resistance storage stability, and in vitro feed dephosphorylation were taken into consideration.

### 7.1. The Effect of pH on the Activity and Stability

This review examines the enzymatic evaluation of the stability of microbial phytase and the desirability of an optimal pH in relation to average transit time and pH in various digestive tract segments of broiler chickens, as reported by Ravindran [69]. The effect of pH on the activity and stability of the desirable crude phytase are presented in Table 5. From optimum pH profiling, the maximum activities of the phytase of *Rhizoctonia* sp. (3 FTU/mL) and *Fusarium verticillioides* (6 FTU/mL) were observed at pH 4.0 and 5.0, respectively, after 30 min incubation of reaction mixture. The phytase of *F. verticillioides* maintained ≥50% activity between pH 4.25 to 5.5 [24]. This finding suggests that the phytase may withstand the pH of the crop. Immobilised phytase of *A. foetidus* MTCC 11682 was found stable over pH 2.5 to 7.5. It showed the highest stability at pH 5.5 at which the activity of 47 FTU/mL was observed [22]. This finding suggests the phytase can be stable in the pH of upper GIT of poultry despite the preincubation time was less than the average digesta retention time.

Crude phytase was produced by using *S. communes* LPB 101, and the effect of pH on the phytase activity was tested over 1.0 to 10.0. The phytase exhibited the highest activity (approximately 165°FTU/dgs) at pH 5.0 maintaining ≥50% of the maximum activity at pH 5 to 8.5. After 24 h storage in pH 2 to 6 at 4°C, the phytase showed activity of 65 to approximately 77.75°FTU/dgs [26]. The finding of optimum profiling implies that the phytase may withstand the pH of the crop. In the same study, with limited information on the temperature as the activity was assayed at 4°C, pH stability is appropriate for feed supplementation. The pH stability of phytase of *Muscodor* sp. UBSX was evaluated after storage in 100 mM citrate buffer (pH 3.0, 4.0, 5.0, and 6.0) at 25°C for 1 h. At pH 5.0, it exhibited 7.36 FTU/mg (100% relative specific activity). However, at pH 4.0 and 6.0, the phytase activity was 25% and 35%. The phytase activity was fully inhibited at pH 3.0 [23]. These findings suggest that the phytase is stable enough in the crop of poultry.

When preincubated in pH 2.5 to 10.0 for 1 h, the crude phytase of *K. marxianus* maintained ≥ 50% of maximum activity. The highest activity of 3 FTU/mL was obtained at pH 4.0 [25]. Phytase of *A. niger* NT7 was observed to be active at a broad range of pH and was found optimal at pH 2.6 and 4.8. The relative activity of ≥50% was maintained at pH 2 to 7 [11]. The findings of Pires et al. and Kumari and Bansal suggest that the phytase under the study might be efficient for poultry feed supplementation as it can withstand the physiological pH of crops, proventriculus, and gizzard.

The effect of pH on the activity and stability of partially purified phytase are presented in Table 6. Phytase of the thermophilic fungus, *Rhizomucor pusillus*, showed an optimum pH of 5.4 and ≈80% activity retained over a wide pH range, 3 to 8 [9]. The phytase of *A. niger*, NCIM 563, showed two pH optima, i.e., 2.5 (Phy I) with 41.47 FTU/mL maximum activity and 4.0 (Phy II) with 10.71 FTU/mL maximum activity. In the same study, the phytase was found stable at pH 1.5 to 3.5 when preincubated at pH 1.5 to 7.0 for 18 h at 4 C and assayed at pH 2.5. Phytase was stable from pH 1.5 to 7.0 when activity was assayed at pH 4.0 [68]. Bala et al. [28] found an optimum pH 5.0 at which phytase activity from *H. nigrescens* BJ82 was taken as 100%, and the phytase maintained ≥50% relative activity from pH 4 to 6.

The pH profiling for phytase from *A. foetidus* MTCC 11682 showed pH 3.5 and 5.5 optima. At pH 5.5, the activity of phytase was 1.5-fold higher compared to its activity at pH 3.5, and this activity was considered 100%. Like pH optimum determination, the phytase exhibited two peaks of maximum stability at pH 3.5 and 5.5. At pH 5.5, the enzyme retained 90% of its original activity even when preincubated for 6 h. Half of the phytase activity was lost at pH 2.5, 4.5, and 6.5. A decline in the activity was observed at pH 7.5 [29]. The phytase activity of *Serratia* sp. PSB-15 and *E. cloacae* PSB-45 showed the maximum activity of 0.13 FTU/mL and 0.07 FTU/mL at pH 6.0 and 7.0, respectively. The phytase maintained ≥50% of the original activity from about pH 2.35 to 7.75 (*Serratia* sp. PSB-15) and about 2.75 to 7.6 (*E. cloacae* PSB-45) [12]. Phytase of *P. acidilactici* SMVDUDB2 showed 4.5 FTU/mL maximum at pH 5.5 and ≥50% activity was maintained over pH 3 to 8.5 [13]. The phytases under the above studies might act efficiently for phytate degradation under pH range in the upper gastrointestinal tract of the broiler [69].

### 7.2. The Effect of Temperature on the Activity and Stability

The desirability temperature optimum and stability of the reviewed microbial phytase was enzymatically evaluated comparing with a body temperature of chicken which is 41 to 42°C [70]. Furthermore, desirability temperature optimum and stability was evaluated in line with commonly used steam conditioning temperature of 65 to 90°C for 15 s duration [41, 42]. Steam conditioning and pelleting process of poultry feeds prevent microbial infection [71] and increase body weights [72].

The effect of temperature on the activity and stability of crude phytase are presented in Table 7. The crude phytase of *Rhizoctonia* sp. and *F. verticillioides* showed the activity of 1.25 FTU/mL and 2.5 FTU/mL, respectively, at a temperature of 50°C. The phytase of *F. verticillioides* maintained ≥50% of the maximum activity at 28 to 60°C. At 80°C, almost all phytase activity was lost [24]. The investigation of the effect of temperature on the phytase of *S. communes* LPB 101 over 30 to 90°C at 10°C interval resulted in an optimal temperature of 50°C [26]. The crude phytase of *Muscodor* sp. UBSX showed complete thermostability at 40°C for 2, 5, 10, 15, 20, 30, 45, and 60 min preincubation time. However, the phytase thermostability was reduced by half at 50 and 60°C after 10 min and 1.5 min preincubation, respectively [23]. The phytase of *A. foetidus* MTCC 11682 showed maximum activity at 37°C. At 70°C, 74% of the activity was retained after 30 min incubation [22].

In optimum temperature profiling, crude phytase of *K. marxianus* presented approximately 3.25 and 3.5 FTU/mL maximum activity at 60 and 80 °C, respectively, showing the presence of more than one type of phytase. However, thermostability investigation at 80 °C resulted in 40% residual phytase activity after 5 to 60 min preincubation. This paradox might be happened due to the assay performed at pH 7.5 at which the phytase had shown the lowest activity [25]. Phytase of *A. niger* NT7 exhibited activity from 40 to 80°C. The optimum temperature was 60°C at which activity was considered 100%. Interestingly, the phytase was found more stable at 50°C with a half-life (*t*_1/2_) of 240 min. It showed a *t*_1/2_ of 120 min at 60°C, but preincubation at 70°C for 60 min resulted in less than half of relative activity [11]. The findings of Pires et al. [25] and Kumari and Bansal [11] indicated phytases understudy might be stable at feed-pelleting temperature and exposure time [12].

The effect of temperature on the activity and stability of partially purified phytase are presented in Table 8. The phytase of *R. pusillus* was found optimally active at 70°C [9]. Soni and Khire [68] determined the optimum temperature for phytase of *A. niger* NCIM 563 over the range of 40 to 65°C. The optimum temperature was 60°C for both Phy I (pH 2.5 active) and Phy II (pH 4.0 active). Thermal stability was investigated via phytase preincubation for 60 min. At 65°C, phytase was active at pH 2.5, retained 80% of its original activity after 15 min but at 70°C activity decreased sharply with only 40% of its original activity remained after 15 min. For Phy II, only 40% of the original activity was retained after 15 min exposure at 65°C, and 36% of the original activity was detected after 5 min exposure at 70°C. Interestingly, Phy I retained 65% of its original activity after 10 min at 70°C, and Phy II retained 60% of its original activity after 10 min at 65°C. The optimum temperature for the activity of phytase of *H. nigrescens* BJ82 was 50°C at which relative activity of the phytase was considered 100%. The phytase showed ≥50% relative activity from approximately 43 to 65°C [28]. Phytase of *A. foetidus* MTCC 11682 exhibited activities in the range of 4 to 80°C with an optimum activity at 37°C. The activity of enzymes did not change much between 37 and 50°C. Thermostability profiling in terms of maintaining the residual activity indicated that the enzyme was stable at 37°C preincubation for 30 min without losing activity, whereas the activity retention was 87% at 50°C preincubation. Interestingly, about 56% of the residual enzyme activity was retained at 80°C preincubation after 30 min exposure [29].

Phytase from *Serratia* sp. PSB-15 was optimally active at 50°C and stable at 70 and 80°C after 20 min preincubation. After treating this phytase, 81.1 and 69.8% of the initial activity was retained, respectively. In the same study, phytase from *E. cloacae* PSB-45 had the maximum phytase activity at 70°C, stable at 70 and 80°C after 10 min preincubation, and showed 52.2 and 18.3% residual activity after treatment at 80°C for 10 and 20 min, respectively [12]. The optimum temperature of phytase of *P. acidilactici* SMVDUDB2 was 37°C. Thermostability assay of the phytase was determined by preincubating the phytase at 50°C, 60°C, 70°C, and 80°C for 3 h. The phytase exhibited good thermostability, and the reduction of the original activity was 25.57% and 32.6% after 3 h at 50°C and 60°C, respectively. Interestingly, 100%, 80%, and 60% residual activities were maintained after 30 min preincubation at 50 and 60°C, 70°C, and 80°C, respectively [13]. The studies suggest that phytases understudy may function under poultry physiological temperature and withstand animal feed pelleting temperature range.

### 7.3. The Effect of Metal Ions on the Activity of Phytase

The effect of various metal ions on the crude phytases is shown in Table 9. Salmon et al. [26] reported that the activity of crude phytase of *S. communes* LPB 101 was stimulated in the presence of 1 mM of K^+^, Ca^2+^, Mg^2+^, Mn^2+^, Zn^2+^, Cu^2+^, and Na^+^. At the same concentration, however, Alves et al. reported that the phytase of *Muscodor* sp. UBSX was inhibited by Na^+^ and Cu^2+^. At both 2 and 5 mM final concentrations, Ca^2+^, Cu^2+^, and Mg^2+^ increased the activity of crude phytase of *K. marxianus* [25]. In another study, the phytase activity of *Aspergillus niger* NT7 was augmented by Ca^2+^ and Zn^2+^, whereas it was inhibited by Mn^2+^, Mg^2+^, and Cu^2+^ at both 1 mM and 5 mM final concentration [11].

The effects of mineral ions on the activity and stability of partially purified phytase were reported [12, 68]. In the investigation of Soni and Khire [68], the effect of metal ions at 1 mM final concentration on the phytase of *A. niger* NCIM 563 was addressed. The phytase retained 79% of its activity at pH 4.0 in the presence of Zn^2+^ while at pH 2.5, it retained 61%. Mg^2+^, Mn^2+^, Ca^2+^, and Fe^3+^ had a stimulatory effect at pH 4.0 while Fe^2+^ and Cu^2+^ had a stimulatory effect at pH 2.5. Zn^2+^ and Cd^2+^ had more inhibitory effects at pH 2.5 than at pH 4.0. Kalsi et al. [12] investigated the effect of metal ions at 5 mM concentration on the phytase of *Serratia* sp. PSB-15 and *E. cloacae* PSB-45. After 30 min preincubation, the relative activity profile with ferrous sulphate was about 50% for PSB-15 phytase, while for PSB-45 phytase, it was 35.8%. With copper sulphate, PSB-15 showed 78% relative activity while PSB-45 phytase was least stable. Manganese sulphate caused more inhibition of PSB-45 phytase. Magnesium sulphate inhibited both phytase activities more than 50%.

## 8. Specificity and Affinity of Phytase to Target Substrate

**Figure 3:**
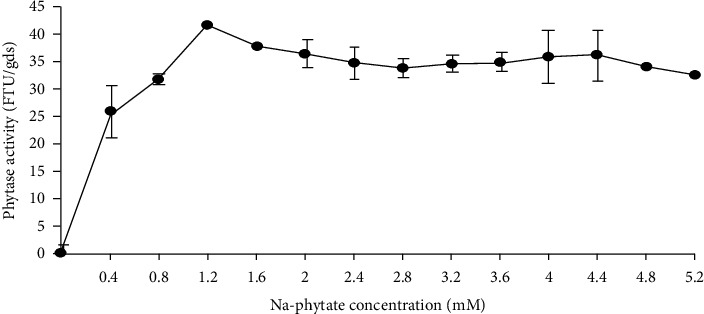
Phytase kinetics of crude phytase of *S. communes* LPB 101 to sodium phytate. The values of phytase activity production in units of enzyme per gram of dry substrate (U gds^−1^) [26] were expressed in the same common unit FTU/gds.

Broad substrate specificity and highest affinity (Michael–Menten constant, *K*_*M*_) toward a target substrate is a required characteristic of phytase for poultry feed addition [31]. The crude phytase of *R. pusillus* showed broad substrate specificity catalyzing the hydrolysis of riboflavin phosphate, AMP, ADP, ATP, paranitrophenol phosphate, NADPH2, phenyl phosphate, and phosphoenol pyruvate, all at concentrations of 5 mM [9]. Salmon et al. [26] reported the affinity of crude phytase of *S. communes* LPB 101 to sodium phytate, 0.4 to 5.2 mM. The *K*_*M*_ of 0.16 mM and maximum velocity (*V*_max_) of 2.087 *μ*mol·mL^−1^ min^−1^ were obtained at 1.2 mM Na-phytate concentration (Figure 3).

Soni and Khire [68], Kalsi et al. [12], and Bhagat et al. [13] reported the affinity of partially purified phytase to sodium phytate. The determined affinity (*K*_*M*_) for Phy I and II of *Aspergillus niger* NCIM 563 was 3.18 and 0.514 mM, respectively, while *V*_max_ was 331.16 and 59.47 *μ*mols/min/mg [68]. Using 0.25 to 10.0 mM Sodium phytate, Kalsi et al. obtained 1.25 and 0.48 m values of *K*_*M*_, and 0.157 and 0.140 U/mL values of *V*_max_ for the phytase of *Serratia* sp. PBS-15 and *E. cloacae* PBS-45, respectively. In the determination of *K*_*M*_ and *V*_max_ of the phytase of *P. acidilactici* SMVDUDB2, different concentrations of sodium phytate (0.1 to 1.8 mM) at pH 5.5 were used resulting in *K*_*M*_ and *V*_max_ values of 0.385 mM and 4.965 *μ*mol/min, respectively [13].

### 8.1. Phytase Resistance to Protease

Proteases are enzymes that can be synthesized in the GIT and that can be classified into six groups based on their catalytic mechanisms: aspartic, glutamic, metalloproteases, cysteine, serine, and threonine proteases [74]. To be used as poultry feed additive, the exogenous phytase must be able to withstand the activity of poultry GIT proteases [75]. In one study Bhagat et al. [13], partially purified phytase of *P. acidilactici* SMVDUDB2 (10 *μ*g/mL) was incubated in 0.2 M glycine-HCl buffer (pH 2.5) containing pepsin (10 *μ*g/mL) in the ratio of 1 : 1, and 0.2 M Tris-HCl buffer (pH 8.0) containing trypsin (10 *μ*g/mL) in the ratio of 1 : 1 at 37°C for a period of 3 h. As a control, the phytase was incubated in the same condition as stated above but lacking pepsin or trypsin. The phytase had strong proteolytic resistance towards pepsin and trypsin. Above 80% of phytase activity was retained after 1 hour incubation period in pepsin and trypsin solution, which reduced to 58.21% and 51.81% after 3 h preincubation in the solutions.

### 8.2. Phytase Storage Stability

Once produced, storage and distribution of feed phytase is a must before it is mixed with animal feed, therefore, phytase stability during storage must be evaluated [16]. Salmon et al. [26] stored crude phytase of *S. communes* LPB 101 at room temperature (26 ± 2°C), cooling (4°C), and freezing temperatures (−18°C) for 5 months. The phytase showed great stability at room and cooling temperature storage, losing only less than 10% activity within the 42 days of storage. The best shelf life was observed at a cooling temperature with 38% of its initial activity maintained after 112 days of storage and still presenting enzymatic activity after 125 days of storage. Ajith et al. [22] stored three phytase samples of *A. foetidus* MTCC 11682 at −20°C. The phytases showed nearly similar stable retention of the enzymatic activity over a period of one year.

Storage stability of partially purified phytase was reported by Kalsi et al. [12]. In the investigation, % residual activity compared to fresh phytase was used for stability evaluation for 30 days of storage at refrigerator (4°C) or room temperature. Phytase from *Serratia* sp. PSB-15 was found highly unstable at room temperature (5.6% residual activity). However, the phytase revealed very good stability in the refrigerator (96.5% residual activity). The authors concluded that the phytase of the isolates could not be stored for longer or if needed, and alternate strategies must be adopted for long-time storage of PSB phytases.

## 9. Dephytinization of Monogastric Animal Feed

**Figure 4:**
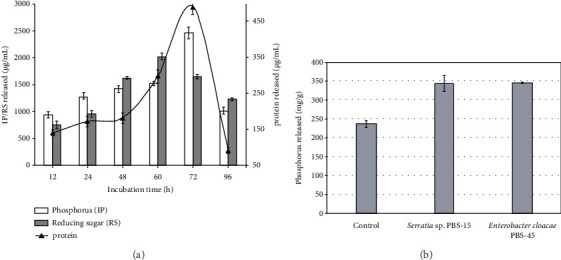
Dephytinization of animal feed ingredient using desirable microbial phytase: (a) release of nutritional components through dephytinization of wheat bran using crude phytase of *A. niger* NT7 [11]. (b) Soybean meal digestion ability of partially purified phytase of *Serratia* sp. PBS-15 and *E. cloacae* PBS-45 [12].

In vivo studies of the digestive process are long, expensive, and difficult to rationalize, whereas in vitro systems may give more accessible insight into parts of this process [76]. As such, feed dephytinization in simulated monogastric animals' digestive system should be conducted for enzymatic evaluation of the produced phytase as poultry feed additive. Kumari and Bansal [11] reported dephytinization activity of crude phytase in which crude phytase (10 FTU) of *A. niger* NT7 was evaluated for dephytinase activity on wheat bran (10 g) under experimental conditions of 100 mM acetate buffer, pH 4.8 at 50°C. The result showed a gradual increase in the release of inorganic phosphorous (2460 ± 102 *μ*g/mL) and proteins (491 ± 20 *μ*g/mL) up to 72 h followed by rapid decline. The liberation of reducing sugars (2019 ± 61 *μ*g/mL) was found increasing until 60 h and later declined gradually (Figure 4(a)). According to the authors, the reason for the decline attributed to plausibly due to end-product inhibition, limitation of substrates, and denaturation of the phytase.

Dephytinization activity of partially purified phytase was reported by Bala et al. [28] and Kalsi et al. [12]. Partially purified phytase of *H. nigrescens* BJ8 was evaluated at 10 grams of wheat and gram flour to 10 FTU of the phytase. Wheat and gram flours were efficiently dephytinized with the concomitant liberation of inorganic phosphate. There was progressive increase in the liberation of inorganic phosphate and soluble protein with an increase in reaction time [28]. Partially purified phytase from *Serratia* sp. PBS-15 and *E. cloacae* PBS-45 was evaluated for their ability to catalyze hydrolysis of soybean meal phytate. The use of 250 FTU/kg of each phytase in an incubated feed mixture at 37°C for 2 h resulted in increased phosphorus compared to the control (Figure 4(b)). The liberated phosphate was in the range of 45 to 46% [12].

## 10. Poultry Feed Phytase Market Size

**Figure 5:**
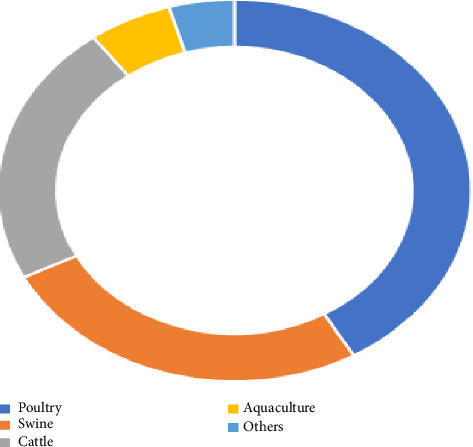
Comparison of animal feed phytase market segments by livestock segment, 2030 estimation [79].

Every year, there is an increase in demand for meat and eggs. As a result, the world's largest and fastest-growing agro-based production sector is now the poultry industry. Phytase supplements for poultry feed are in higher demand due to the cost of associated feed and the pollution of the environment caused by phosphorus [76–78]. The poultry feed phytase market was estimated to be worth US $210 million in 2021 and is projected to grow at a compound annual growth rate (CAGR) of 6.0% in terms of revenue over the forecasting period, 2022–2030, according to a September 2022 Global Marker Insight report [79]. The livestock feed phytase market segment in the report included the markets for poultry, pigs, cattle, aquaculture, and other animals. Of these, the poultry feed phytase market segment is expected to lead the global market (Figure 5).

The worldwide market will be dominated by the powdered or granular phytase market segment. In addition to holding a combined market share of more than 60%, they are anticipated to grow at the fastest rate in terms of revenue between 2018 and 2026 [80]. For them, the revenue trend for the 2022–2030 forecasting period showed the same pattern. This market for liquid phytase is predicted to expand at a compound annual growth rate (CAGR) of 6.5%. Because liquid phytase ensures optimal post-feed-pelleting application and keeps heat-sensitive feed phytase from denaturing, it is in high demand [79].

## 11. Regional Outlook of the Feed Phytase Market

**Figure 6:**
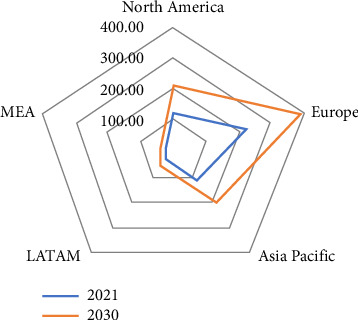
Animal feed phytase market by region segment (US $ million) in 2021 and 2030. MEA represents Middle East and Africa. LATAM stands for Latin America [79].

North America, Europe, Asia Pacific, Latin America, and the Middle East and Africa (MEA) are the traditional regions into which the feed phytase market is divided [79–83]. The Asia-Pacific region is currently experiencing a notable surge in per capita income and rapid economic growth. The demand for dairy products, meat, and eggs increased as a result of these factors. As a result, the livestock industry in the area has grown, necessitating careful consideration of feed additives such as phytase to enhance animal nutrition [79]. However, a high feed phytase cost will limit the market size in developing nations (Figure 6). The complicated biotechnological process of producing phytase necessitates a large investment in R&D as well as manufacturing, which accounts for the high cost [81].

The countries that are preferred, according to the Global Market Insight report [79], are North America's USA and Canada; Europe's Germany, UK, Spain, Russia, France, and Italy; Asia Pacific's China, India, Japan, Australia, Malaysia, Thailand, and Indonesia; Latin America's Brazil, Mexico, and Argentina; and Middle East and Africa's Saudi Arabia, UAE, and South Africa. North America is anticipated to lead the global feed phytase market from 2023 to 2030, driven by the region's well-established livestock industry, which includes swine, poultry, and cattle farms, according to a Business Research Insights report updated on October 2023 [81]. Nonetheless, the European animal feed phytase market is expected to gain dominance in the same forecast year and reach a valuation of US $390 million by 2030, according to Global Market Insight [79]. The market participants that were profiled during the studies may be the cause of the disparity in feed phytase market analysis trends (Table 10). VTR BioTech was profiled by Business Research Insights [81], but not by Global Market Insight [79]. Alltech, Novozymes, and Novus International were profiled in Global Market Insight [79], but they were not in Business Research Insights [81].

## 12. Regulatory Guidelines for Microbial Phytase Supplementation to Poultry Diet

Since the particular experimental protocol used defines the enzyme activity, there is no set regulatory guideline for the use of phytase in animal feed [79, 84]. Phytase-based feed additives are permitted under the category of zootechnical additives and functional group of digestibility enhancers as enacted in article 6 and Annex 1 of regulation (EC) No 1831/2003 on additives for use in animal nutrition, which was ratified in the European Union (EU) [85]. The International Organization for Standardization (ISO) and European Standards (EN) standard 30024 provides a standardized procedure for phytase activity measurement and is appropriate for the official management of a class of phytase products. In order to screen feed samples supplemented with the 4a16 phytase, EN ISO Standard 30024 was developed because, unfortunately, it is not appropriate for determining the phytase activity of a new feed additive encoded as 4a16 [84].

Some of the phytase feed additives that have been approved by the European Food and Safety Authority (EFSA) in accordance with regulations are listed in Table 11. One of the requirements for authorization, as stated in the regulation, is that no feed additive has a negative impact on the environment, animal health, or human health [85].

The Center for Veterinary Medicine at the Food and Drug Administration (FDA) in the United States ensures a secure supply of animal feed. The center reviews submissions for premarket animal food ingredients, approving safe food additives for use in animal food, assessing generally recognized as safe (GRAS) notices, acting as the scientific reviewer for feed ingredient definitions defined by the Association of American Feed Control Officials (AAFCO), examining notifications regarding biotech plants, and examining labels for pet foods and animal food products with specific claims, among other duties (https://www.fda.gov/animal-veterinary/products/animal-food-feeds). Supplementary Table 1 lists notified poultry feed phytases that are generally recognized as safe (GRAS).

## 13. Conclusions and Recommendations

In isolation, screening and identification of native microbes for phytase production for poultry, the selection of phytate enriched source environment, the simultaneous use of both agar and broth PSM, and the use of cultural, morphological, biochemical, and molecular methods for isolate identification are very crucial. Fungal phytase is better produced by solid-state fermentation, while bacterial phytase is better produced by submerged fermentation. The economics of producing phytase is significantly impacted by the use of substrates made from agricultural or agroindustrial waste. The phytase that is produced has been assessed for its enzymatic suitability as an additive for poultry feed based on one or more biochemical properties. The need to improve the digestibility of poultry feed through phytase supplementation stems from the high demand for an animal protein diet. As a result, the market for poultry feed phytase is expected to grow at a compound annual growth rate (CAGR) of 6.0% between 2022 and 2030. The cost of the biotechnological process used to produce engineered phytase is causing developing nations to fall behind in the phytase field. Standards to assess the quality and safety of phytase feed additives for poultry have been ratified in response to the growth of phytase-producing businesses, despite the difficulty in standardizing the process.

Therefore, by extracting crude or partially purified phytase from native microbial culture filtrate, researchers should determine whether using phytase as an additive in poultry feed is feasible in developing nations. The pH value specific to the intended breed of poultry, the pepsin secreted from the poultry's GIT, the temperature and exposure duration of the feed pelleting process, the particular feed storage conditions, the stability of the feed's common mineral composition, and the in vitro feed hydrolysis in a reaction mixture that simulates the GIT are all necessary to evaluate this product. It is necessary to take into account hot, acidic, and plant litter-containing environments when isolating and screening appropriate phytase-producing microbes. Researchers and nutritionists should collaborate to create and assess chicken diets that have the right amount of microbial phytase. It is recommended that poultry farmers consult researchers or nutritionists when choosing phytase feed additives for their particular breed of birds. If not, manufacturers ought to share the approval documents for phytase feed additives since they make explicit the usage guidelines, the intended animal, and the minimum level of inclusion.

## Figures and Tables

**Table 1:** Screening and identification of potential microbial isolates that produce desirable phytase.

Potential isolate	Source of the isolate	Size of CZ (mm)	Phytase activity (FTU/mL)	Identification	Reference
*Aspergillus foetidus* MTCC 11682	Soil	NA	NA	NA	[22]
*A. niger* NT7	Rhizospheric soil of maize	1.5	0.19	Microscopic examination and 18S rRNA gene sequencing	[11]
*Aspergillus* sp. FS3	Soil	NA	51.53^b^	NA	[10]
*Enterobacter cloacae* PSB-45	Compost	0.6	0.305	16S rRNA gene sequencing	[14]
*Escherichia coli* (RS15)	Rhizospheric soil of lentil	30	0.071	Morphological, biochemical, and 16S rRNA sequencing	[35]
*Kluyveromyces marxianus*	NA	NA	0.06	NA	[25]
*Klebsiella pneumoniae* (RS8)	Rhizospheric soil of lentil	32	0.054	Morphological, biochemical, and 16S rRNA sequencing	[35]
*Muscodor* sp. (UBSX)	Shoot of *Coffea arabica* L.	4.41^a^	NA	ITS1 and ITS4 region sequencing	[23]
*Pediococcus acidilactici* SMVDUDB2	Cheese	8	5.18	16S rRNA gene sequencing	[15]
*P. acidilactici* BN5B	Neonatal faeces	NA	4.55	Cultural, MALDI-TOF MS, and 16S rRNA sequencing	[36]
*Pichia anomala* UDDM-55	Flower bud of *W. fruticosa*	NA	0.006	Morphological, physiological, and biochemical test	[37]
*Pseudomonas aeruginosa* KBM2	Soil	5^a^	0.381	Morphological and 16S rRNA sequencing	[38]
*Rhizomucor pusillus*	Composting soil	NA	NA	NA	[9]
*Serratia* sp. PSB-15	Soybean rhizospheric soil	3.6	0.285	16S rRNA gene sequencing	[14]
*Staphylococcus* sp. BAM 8	Kali	38	0.37	Morphological and biochemical tests	[12]
*S. lentus* ASUIA279	Endophyte of maize root	NA	1.913	Morphological, Gram stain, catalase test, API staph, and API 50 CH	[13]

^a^The value is enzyme index (EI). ^b^FTU/gram of dried substrate (gds) produced through SSF. NA: not available; CZ: clear zone. The phytase activity units are uniformly converted to FTU/mL for phytase produced through SmF.

**Table 2:** Phytase screening medium (PSM) (g/L).

Ca-phytate	Na-phytate	Glucose	NH_4_NO_3_	KCl	CaCl_2_	MgSO_4_·7H_2_O	MnSO_4_·4H_2_O	FeSO_4_·7H_2_O	ZnSO_4_·7H_2_O	Agar	References
—	10	—	2	0.5	—	0.5	—	0.05	0.1	19	[10]^2b^
1	—	15	2	0.5	—	0.5	0.3	0.3	—	15	[24]
—	4	20	5	0.5	2	0.5	0.01^a^	0.01	—	15	[17]
3	—	15	5	0.5	—	0.5	0.01	0.01	—	20	[28]
—	—	—	—	0.5	0.66	0.5	—	0.12	0.17	30	[23]^3b^
4	—	10	2	0.5	—	0.5	0.01	0.01	—	15	[14]
—	1	15	2	0.5	—	0.5	0.3	0.3	—	20	[15]
5	—	15	5	0.5		0.5	0.01	0.01	—	15	[38]

^a^MnSO_4_·7H_2_O; ^2b^the author prepared the medium contained 2 g NaNO_3_; ^3b^the authors prepared the medium contained 5 g/L phytic acid and 3 g NaNO_3_.

**Table 3:** Substrates and fermentation types in production of desirable microbial phytase.

Microbial species	Substrate	Fermentation	Optimum production	Ref.
*Aspergillus flavus* PHY168	Sodium phytate	SmF	3.61 U/mL	[47]
*Aspergillus niger* 7A-1	Triticale	SSF	54.56 U/mL	[48]
*A. niger* CFR 335	Wheat bran, rice bran, and groundnut cake in the ratio of 2 : 1 : 1	SSF	76 U/gds	[49]
*A. niger* CFR 335	Potato dextrose broth	SmF	9.6 U/mL	
*A. niger* F3	Citrus peel	SmF	3.91 U/mL	[50]
*A.niger* NT7	Wheat bran	SSF	208.30 U/gds	[11]
*A. niger* FS3	Citric pulp bran	SSF	0.62 U/mL	[51]
*A. niger* NCIM 563	Wheat bran	SSF	50 U/g dMB	[52]
*Aspergillus tubingensis* SKA	Wheat bran	SSF	60.42 U/mL	[53]
*Bacillus subtilis* DR6	Calcium phytate	SmF	378 U/mL	[54]
*B. subtilis* US417	Wheat bran	SSF	1,180 U/(kg × h)	[44]
*B. subtilis* US417	5% wheat bran	SmF	2,330 U/(kg × h)	[44]
*Enterobacter cloacae* PSB-45	Sodium phytate	SmF	0.305 U/mL	[12]
*Enterobacter sakazakii* ASUIA279	Rice bran	SmF	5.5 *μ*m/L of P	[55]
*Enterobacte*r sp. ACSS	Wheat bran with sodium phytate	SmF	83.2 U/mL for shake flask	[56]
*Escherichia coli* BL21	Glucose	SmF	120 U/mL	[57]
*Klebsiella* sp.	Wheat bran with sodium phytate	SmF	94 U/mL	[58]
*Klebsiella pneumoniae* 9-3B	Sodium phytate sole carbon source	SmF	596 U/mL for cell extract	[39]
*Lactobacillus amylovorus* B4552	Sodium phytate	SmF	125–146 U/mL	[59]
*Lactobacillus reuteri*	Sodium phytate	SmF	200 U/mL	[60]
*Muscodor* sp. (UBSX)	Wheat bran	SmF	4.10 U/mg of protein	[23]
*Neurospora sitophila*	Rice straw powder and soybean curd residue plus 100 ml of salt solution	SmF	8.3 U/mL	[43]
*N. sitophila*	Rice straw powder and soybean curd residue plus 6.0 ml of salt solution	SSF	195.66 U/g	[43]
*Rhizopus oligosporus*	Coconut oil cake	SSF	14.29 U/gds	[61]
*R. oligosporus MTCC556*	Rice bran ADT47	SSF	23.82 U/gds	[62]
*R. oligosporus* MTCC556	Rice bran	SSF	31.3 U/gds	[63]
*Schizophyllum commune* LPB 101	Wheat bran	SSF	113.7 U/gds	[26]
*S. commune* LPB 101	Wheat bran	SSF	113.7 U/gds	[26]
*Sporotrichum thermophile*	Wheat bran and sodium phytate	SmF	126 U/l/h	[64]
*Thermoascus aurantiacus* TUB F 43	3.75% (w/v) wheat bran particle	SmF	152.36 U/mL	[65]
*T. aurantiacus* SL16W	Rice bran	Sem-SSF	58.6 U/gds	[45]
*Thermomyces lanuginosus*	Rice bran	SSF	0.314 U/mL/min	[66]

SSF: solid-state fermentation; semi-SSF: semisolid-state fermentation; SmF: submerged fermentation; gds: gram of died substrate; g dMB: gram of dried moldy bran; Ref.: reference.

**Table 4:** Summary of partial purification steps of desirable microbial phytase.

Purification step	Total protein (mg/mL)	Phytase activity^a^ (FTU/mL)	Specific activity (FTU/mg)	Fold	% yield	Source	References
Crude filtrate	NA	NA	NA	1	100	*A. niger* NCIM 563	[68]^c^
95% (NH_4_)_2_SO_4_	NA	NA	NA	NA	NA		
Sephadex G-25	NA	NA	224	3.0	95		

Crude filtrate	5.580	0.223	0.040	1.000	100	*H. nigrescens* BJ82	[28]^c^
25% (NH_4_)_2_SO_4_	0.283	0.038	0.133	3.325	17.04		
50% (NH_4_)_2_SO_4_	3.960	0.047	0.012	0.3	21.08		
80% (NH_4_)_2_SO_4_	9.630	0.215	0.022	0.55	96.41		

Crude filtrate	NA	NA	NA	1	100	*Serratia* sp.PSB-15	[12]^d^
70% (NH_4_)_2_SO_4_	NA	NA	NA	NA	NA		
Dialysis	NA	NA	NA	2.18	44.63		
Crude filtrate	NA	NA	NA	1	100	*E. cloacae* PSB-45	
70% (NH_4_)_2_SO_4_	NA	NA	NA	2.55	62.30		
Dialysis	NA	NA	NA	2.55	62.30		

Crude filtrate	3600	45400	12.6	1.0	100	*A. foetidus* MTCC 11682	[29]^c^
90% (NH_4_)_2_SO_4_	NA	NA	NA	NA	NA		
Dialysis (10–14 MCO membrane)	245	10200	41.6	3.3	22.5		
Sephacryl S-200HR	20	5879	293.9	23.3	12.9		

Crude filtrate	145	5541	38.21	1	100	*P. acidilactici* SMVDUDB2	[13]^b^
(60–90%) (NH_4_)_2_SO_4_	10.5	1000.5	95.29	2.49	18.06		
HIC	2.04	500.04	245.12	6.42	9.02		

^a^one unit of phytase activity was defined as the amount of phytase that releases 1 *μ*mol phosphate per minute under the assay conditions, ^b^determined protein concentration using the Bradford method, and ^c^determined protein concentration using the Lowry method; ^d^protein concentration determination method was not indicated. HIC: hydrophobic interaction chromatography.

**Table 5:** pH optimum and stability of crude desirable phytase for poultry feed application.

Microbial source	Opti. pH	IT (min) T° (°C)	Max. activity	pH at ≥50% activity	pH stability, max. activity	PIT (h), T° (°C)	Reference	Recommendation
*A. adeninvoran* CBS 8335	4.5 to 5	5 to 20, 70	NA	NA	NA	NA	[21]	In line to crop and proventriculus pH
*F. verticillioides*	5	30, 37	6 FTU/mL	4.25 to 5.5	NA	NA	[24]	In line to crop pH
*S. communes* LPB 101	5	30, 50	≈165 FTU/dgs	5 to 8.5	3 to 9	24, 4	[26]	In line to crop pH
*Muscodor sp.*	NA	NA	NA	NA	pH 5, 7.36 FTU/mg	1, 25	[23]	In line to crop pH
*A. foetidus* MTCC 11682	NA	NA	NA	NA	2.5 to 7.5, at pH 5.5, 47 FTU/mL	0.5, 37	[29]	In line to upper GIT pH
*K. marxianus*	NA	NA	NA	NA	2.5 to 10.0, at pH 4, 3 FTU/mL	1, 4	[25]	In line to upper GIT and duodenum pH
*A. niger* NT7	2.6 or 4.8	30, 50	100% at pH 2.6	2 to 7	NA	NA	[11]	In line to upper GIT pH

Opti: optimum; Max: maximum; IT: incubation time; T°: temperature; PIT: preincubation time; NA: not available; GIT: gastrointestinal tract. ≈ is approximate value. Recommendation provided comparing with crop (pH 5.5, 10 to 50 min transit time), proventriculus/gizzard (pH 2.5 to 3.5, 30 to 90 min transit time), duodenum (pH 5 to 6, 5 to 10 min transit time), jejunum (pH 6.5 to 7.0, 20 to 30 min transit time), ileum (pH 7.0 to 7.5, 50 to 70 min transit time), and cecum/colon (pH 8.0, 20 to 30 min transit time) [69].

**Table 6:** pH optimum and stability of desirable partially purified microbial phytase.

Microbial source	Opti. pH	IT (min), T° (°C)	Max. activity (FTU/mL)	pH at ≥50% activity	pH stability, max. activity	PIT (h), T° (°C)	Reference	Recommendation
*R. pusillus*	5.4	30, 37	100%	3 to 8	NA	NA	[9]	In line to GIT pH
*A. niger* NCIM 563 (PhyI)	2.5	30, 50	41.47	1.75 to 2.75	1.5 to ≈3.5	18, 4	[68]	In line to provent and gizzard pH
*A. niger* NCIM 563 (PhyII)	4	30, 50	10.71	3.75 to 4.75	2 to 7	18, 4		In line to GIT pH
*H. nigrescens* BJ82	5	30, 50	100%	4 to 6	NA	NA	[28]	In line to crop pH
*Serratia* sp. PSB-15	6	30, 37	0.13	≈5 to 8.75	≈2.35 to 7.75, at pH 7, 100%	2, 4	[12]	In line to GIT pH
*E. cloacae* PSB-45	7	30, 37	0.07	≈4.25 to 7.75	≈2.75 to 7.6, at pH 7, 100%	2, 4		In line to GIT pH
*A.foetidus* MTCC 11682	3.5 and 5.5	30, 37	100% at pH 5.5	2.5 to 7.5	2.5 to 6.5, at pH 5.5, 100%	6, 37	[29]	In line to upper GIT, duodenum, and Jejum pH
*P. acidilactici* SMVDUDB2	5.5	60, 37	≈4.5	3 to 8.5	NA	NA	[13]	In line to upper GIT and duodenum pH

Opti: optimum; max: maximum; IT: incubation time; T°: temperature; PIT: preincubation time; NA: not available; GIT: gastrointestinal tract. ≈ is approximate value. I and II stand for phytase I and phytase II, respectively. Recommendation provided comparing with crop (pH 5.5, 10 to 50 min transit time), proventriculus/gizzard (pH 2.5 to 3.5, 30 to 90 min transit time), duodenum (pH 5 to 6, 5 to 10 min transit time), jejunum (pH 6.5 to 7.0, 20 to 30 min transit time), ileum (pH 7.0 to 7.5, 50 to 70 min transit time), and cecum/colon (pH 8.0, 20 to 30 min transit time) [69].

**Table 7:** Temperature optimum and stability of crude phytase for poultry feed application.

Microbial source	Opti. T° (°C)	IT (min), pH	Max. activity	T° (°C) at ≥50% activity	T° stability (°C), max. activity	PIT (min), pH	Reference	Recommendation
*F. verticillioides*	50	30, 5.5	2.5 FTU/mL	28 to 60	NA	NA	[24]	In line to GIT T°
*S. communes* LPB 101	50	30, 5.5	50 FTU/dgs	40 to 60	NA	NA	[26]	In line to GIT T°
*Muscodor sp.*	NA	NA	NA	NA	40, 50, 60	1.5, 5	[23]	In line to GIT T°
*A.foetidus* MTCC 11682	NA	NA	NA	NA	4 to 80, at 37, 50 FTU/mL	30, 5.5	[29]	In line to GIT T° and feed pelleting T°
*K. marxianus*	60 and 80	30, 5	at 80, ≈3.5 FTU/mL	10 to 80	80 (not stable)	80, 7.5	[25]	In line to GIT T° and feed pelleting T°
*A. niger* NT7	60	30, 4.8	at 60, 100%	30 to 70	50 and 60	120, 4.8	[11]	In line to GIT T° and feed pelleting T°

Opti: optimum; max: maximum; IT: incubation time; T°: temperature; PIT: preincubation time; NA: not available; GIT: gastrointestinal tract. ≈ is approximate value. Recommendation provided comparing with 41 to 42°C body temperature of chicken [70] and 65 to 90°C steam conditioning temperature for 15 s duration [41, 42].

**Table 8:** Temperature optimum and stability of desirable partially purified microbial phytase.

Microbial source	Opti. T° (°C)	IT (min), pH	Max. activity	T° (°C) at ≥50% activity	T° stability (°C), max. activity	PIT (min), pH	References	Recommendation
*R. pusillus*	70	30, 5.4	100%	50 to ≈70	NA	NA	[9]	In line to feed pelleting T°
*A. niger NCIM* 563 (PhyI)	60	30, 2.5	100%	≈43.75 to ≈63.75	40 to 65	1, 2.5	[68]	In line to GIT and feed pelleting T°
*A. niger NCIM* 563 (PhyII)	60	30, 4	100%	≈44.75 to ≈63.75	40 to 50	1, 4		In line to GIT and feed pelleting T°
*H. nigrescens*	50	30, 5	100%	≈43 to 65	NA	NA	[28]	In line to GIT and feed pelleting T°
*Serratia* sp. PSB-15	50	30, 6	≈0.085 FTU/mL	30 to ≈59	70 and 80	1/3, 6	[12]	In line to GIT and feed pelleting T°
*E. cloacae* PSB-45	7	30, 6	≈0.069 FTU/mL	≈50 to 80	70 and 80	1/6, 6		In line to feed pelleting T°
*A. foetidus* MTCC 11682	37	30, 5.5	100%	4 to 80	4 to 80, at 37, 100%	1/2, 5.5	[29]	In line to GIT and feed pelleting T°
*P. acidilactici* SMVDUDB2	37	60, 5.5	≈4.2 FTU/mL	≈22 to 60	50 and 60	3, 5.5	[13]	In line to GIT and feed pelleting T°

Opti: optimum; max: maximum; IT: incubation time; T°: temperature; PIT: preincubation time; NA: not available; GIT: gastrointestinal tract. ≈ is approximate value. I and II stand for phytase I and phytase II, respectively. Recommendation provided comparing 41 to 42°C body temperature of chicken [70] and 65 to 90°C steam conditioning temperature for 15 s duration [72, 73].

**Table 9:** The effect of metal ions on the crude desirable phytase for poultry feed application.

Ions	Species	RA^a^ (%)	RA^b^ (%)	RA^c^ (%)	RA^d^ (%)
Control	—	100	100	100	100

Mn^2+^	Sulphate	369.7	NA	NA	NA

K^+^	Sulphate	356.5	NA	NA	NA

Ca^2+^	Chloride	NA	NA	116.0 ± 2.8	273.0 ± 3.1
	Carbonate	NA	NA	144.0 ± 3.1	203.0 ± 1.8
	Sulphate	342.9	NA	204.0 ± 0.5	387.0 ± 9.5

Mg^2+^	Sulphate	315.9	NA	NA	NA
	Chloride	NA	NA	217.0 ± 3.87	268.0 ± 4.6

Fe^3+^	Sulphate	308.4	NA	NA	NA
	Chloride	293.1	NA	96.0 ± 0.3	52.0 ± 1.0

Fe^2+^	Sulphate	NA	86.12	NA	NA

Na^+^	Sulphate	294.8	NA	NA	NA

Ni^2+^	Sulphate	285.4	NA	NA	NA

Cu^2+^	Sulphate	274.4	40.07	310.0 ± 31.3	135.6 ± 3.0

Zn^2+^	Sulphate	211.6	NA	60.6 ± 1.4	32.8 ± 2.5

Co^2+^	Sulphate	120.4	NA	NA	NA

Na^+^	Chloride	102.8	11.75	NA	NA

NH^+^	Molybdate	0	NA	NA	NA
	Chloride	NA	NA	127.0 ± 4.8	238.0 ± 14.1

Al^3+^	Chloride	NA	49.9	NA	NA

Hg^2+^	Chloride	Na	26.97	NA	NA

Ag^2+^	Sulphate	NA	NA	116.7 ± 12.1	289.0 ± 20.6

^a^The effect of metal ions at 1 mM on the activity of phytase of *S. communes* LPB 101 [26]; ^b^the effect of metal ions at 1 mM on the activity of phytase of *Muscodor* sp. USBX [23]; ^c,d^the effect of metal ions at 2 and 5 mM, respectively, on the activity of phytase of *K. marxianus* [25]; RA and NA represent relative activity and are not available, respectively.

**Table 10:** Key phytase market player filed in three phytase market reports.

Key phytase market player companies	Acumen research and consulting [80]	Business research insights [81]	Global market insight [79]
AB Enzymes (Germany)	√	√	√
Adisseo (France)	—	√	√
Advanced Enzymes (India)	—	—	—
Alltech (USA)	—	—	√
BASF (Germany)	√	√	√
Beijing Smistyle (China)	√	—	—
DSM (Netherlands)	√	√	√
DuPont (USA)	√	√	√
Dynavax Gmbh (Germany)	—	—	—
Huvepharma (Bulgaria)	—	—	—
Jinan Tiantianxiang (TTX) Co., Ltd. (China)	√	—	—
Kemin Industries (USA)			
Novozymes (Denmark)	—	—	√
Novus International (USA)	—	—	√
Qingdao Vland Biotech Inc (China)	—	—	—
Willows Ingredients (Ireland)	—	—	—

√ represents profiled key market player companies. The countries in brackets show the place of companies' headquarters.

**Table 11:** Some approved phytases by EFSA for poultry feed additive.

Phytase source and host	Brand name	Product form	Reference
A synthetic gene (NOV9X) encoding 6-phytase expressed in *Pichia pastoris* sNOV9Xpp27	Quantum™ phytase	Liquid (quantum™ phytase 5000 L) and granule (quantum™ phytase 2500 D)	[86]
*Escherichia coli* AppA gene-encoding 6-phytase expressed in *Trichoderma reesei CBS 122001*	Finase® EC	A solid (Finase®EC 40 P), and liquid (Finase®EC 10 L, and Finase®EC 5 L)	[87]
6-phytase gene expressed in *Trichoderma reesei* (CBS 126897)	Quantum® blue	Liquid form (quantum blue 5 L), and quantum blue 10 L, solid and granulated (quantum blue 5 G), and solid (quantum blue 40 P)	[88]
6-Phytase gene expressed in *Trichoderma reesei* CBS 146250	Axtra®PHY GOLD	Liquid (Axtra®PHY GOLD 30 L) and tan granular (Axtra®PHY GOLD 30T and 65G)	[89]
*Buttiauxella* 6-phytase expressed in *Trichoderma reesei* (ATCC SD-6528)	Axtra®PHY 20000 TPT2	Solid granulate	[90]
6-Phytase gene expressed in *Komagataella phaffii* (CGMCC 7.19)	Nutrase P	Powder formulation (Nutrase PD), granulate (Nutrase PG), thermostable granulate (Nutrase PTS), and liquid (Nutrase PL)	[91]
6-Phytase gene expressed in *Komagataella phaffii* (CGMCC 7.370)	VTR-phytase	Liquid/powder	[92]
6-Phytase gene expressed in *A. oryzae* DSM 33699	RONOZYME® HiPhos	Solid (RONOZYME® HiPhos GT) and liquid (RONOZYME® HiPhos L)	[93]

## Data Availability

Data sharing is not applicable to this article.

## Supplementary Materials

Supplementary Materials Supplementary Table 1: notified poultry feed phytases that are generally recognized as safe (GRAS). Select the AGRN number for detailed count of each feed phytase.
