# Supplementary material for: Review on Desirable Microbial Phytases as a Poultry Feed Additive: Their Sources, Production, Enzymatic Evaluation, Market Size, and Regulation
Source: Int J Microbiol. 2024 Jun 7;2024:9400374. doi: 10.1155/2024/9400374 (PMC11221984; doi:10.1155/2024/9400374)
Supplement: Supplementary Materials — Supplementary Table 1: notified poultry feed phytases that are generally recognized as safe (GRAS). Select the AGRN number for detailed count of each feed phytase. [file 9400374.f1.docx]

| AGRN (select AGRN No. for detailed record) | Notifier | Substance | Intended Use | Intended Species | Date of Filing | FDA's Letter (select to view letter) |
| --- | --- | --- | --- | --- | --- | --- |
| [14](https://www.fda.gov/files/UCM352512.pdf) | DSM Nutritional Products | Phytase enzyme produced by an *Aspergillus oryzae*strain expressing a synthetic gene coding for a 6-phytase from *Citrobacter braakii* | To increase the digestibility of phytin-bound phosphorous or to increase phosphorous availability from phytate in poultry diets when fed at the rate of 250-4000 FYT/kg feed. | Poultry (turkey, broiler chickens, and egg laying hens) | 11/14/2012 | [FDA has no questions. (PDF - 3 pages)](https://www.fda.gov/media/86111/download?attachment) |
| [21](https://www.fda.gov/files/combined%20redacted%20notice%20AGRN%2021.pdf) | Agrivida, Inc. | Ground grain obtained from a corn (*Zea mays*) variety that expresses an altered *appA* 6-phytase gene obtained from *Escherichia coli* strain K12 (transformation event PY203) | To increase the digestibility of phytin-bound phosphorous or to increase phosphorous availability from phytate in poultry feeds when used at a rate of 75 g to 1.7 kg per ton of complete feed and providing 250-6000 phytase units (FTU)/kg complete feed. | Poultry | 7/28/2016 | [FDA has no questions. (PDF – 4 pages)](https://www.fda.gov/media/108194/download?attachment) |
| [32 (PDF - 105 pages)](https://www.fda.gov/media/140655/download?attachment) | Agrivida, Inc. | Ground grain obtained from a corn (*Zea mays*) variety that expresses an altered *appA* 6-phytase gene obtained from *Escherichia coli*strain K12 (transformation event PY1203) | To increase the digestibility of phytin-bound phosphorous or to increase phosphorous availability from phytate in swine feeds when used to provide 500-4500 phytase activity units (FTU)/kg complete feed, or poultry feeds when used to provide 250-6000 FTU/kg complete feed. | Swine and poultry | 7/24/2019 | [FDA has no questions. (PDF - 4 pages)](https://www.fda.gov/media/137127/download?attachment) |
| [39 (PDF-594 pages)](https://www.fda.gov/media/151494/download?attachment) | BASF Enzymes LLC | Phytase enzyme produced by *Pseudomonas fluorescens* strain BD50104 expressing an altered*appA*6-phytase gene from *Escherichia coli* strain K12 | To increase the availability of phytin-bound phosphorus in poultry diets at 250-2000 U/kg in complete feed. | Poultry | 7/14/2020 | [At notifier’s request, FDA ceased to evaluate the notice. (PDF - 1 page)](https://www.fda.gov/media/148936/download?attachment) |
| [55 (PDF - 595 pages)](https://www.fda.gov/media/168124/download?attachment) | BASF Enzymes LLC | Phytase enzyme produced by *Pseudomonas fluorescens* strain BD50104 expressing an altered *appA* 6-phytase gene from *Escherichia coli* strain K12 | To increase the availability of phytin-bound phosphorus in broiler diets at 500-2,000 U/kg in complete feed. | Broiler chickens | 1/20/2022 | [FDA has no questions. (PDF - 4 pages)](https://www.fda.gov/media/161638/download?attachment) |
